# Application of hs-CRP in Assessment of Tissue Inflammation Following Implantation of Biodegradable Polymer in Experiment

**DOI:** 10.3390/ijms252011183

**Published:** 2024-10-17

**Authors:** Igor A. Eisenach, Galina A. Lapii, Alexandra K. Uzyumova, Elena L. Lushnikova, Victor S. Ovchinnikov, Anastasia O. Solovieva, Vasiliy A. Naprimerov

**Affiliations:** 1 Institute of Molecular Pathology and Pathomorphology, Federal Research Center for Fundamental and Translational Medicine, 2 Timakova St., Novosibirsk 630117, Russia; galapii@frcftm.ru (G.A.L.); apichigina@yandex.ru (A.K.U.); ellushnikova@frcftm.ru (E.L.L.); 2 Research Institute of Clinical and Experimental Lymphology, Branch of the Institute of Cytology and Genetics, Siberian Branch of Russian Academy of Sciences, 6 Arbuzova, Novosibirsk 630060, Russia; ovch.v.s@mail.ru (V.S.O.); solovevaao@yandex.ru (A.O.S.); 3 The Federal Research Center Institute of Cytology and Genetics, Siberian Branch of Russian Academy of Sciences, 10 Prospekt Lavrentyeva, Novosibirsk 630090, Russia; nva61@bionet.nsc.ru

**Keywords:** biodegradable polymer, implant, tissue reaction, inflammation, bioinertness, highly sensitive C-reactive protein (hs-CRP)

## Abstract

Implants made of biodegradable polymers are replaced by regenerating tissues through inflammation. The changes occurring in tissues and the organism are of practical interest for studying the biocompatibility of the material and searching for systemic markers in the blood that reflect inflammation in peri-implantation tissues. The highly sensitive C-reactive protein (hs-CRP) measurements in blood and morphometric studies of tissue surrounding the implant were carried out in the experiment within three months of implantation of a biopolymer consisting of polycaprolactone (PCL) and polytrimethylene carbonate (PTMC). During the first month, tissue inflammation decreased, and the blood level of hs-CRP did not increase. The polymer biotransformation began in the tissues after a month of implantation and was accompanied by inflammation moving deeper into the matrix. Proliferation of inflammatory cells in tissues was reflected in an increase in the hs-CRP level three months after polymer installation. The result achieved confirmed the polymer’s bioinertness. The level of hs-CRP in the blood of the animals correlated with the level of inflammation in peri-implantation tissues, reflecting the activity of inflammation in the process of polymer biotransformation. This inflammation protein can be recommended for assessing tissue processes following implantation of biopolymers and their biocompatibility.

## 1. Introduction

Currently, various synthetic polymers are used in surgery in almost every operation as prostheses or suture material [1,2]. Recently, a new area of development in implant materials has appeared: biodegradable polymers [3,4]. Their attractiveness lies in their ability to completely degrade and be replaced by the patient’s own tissues [5]. The degradation process includes natural chemical and physical decomposition into polymer chains and biotransformation involving cells, primarily macrophages which absorb these polymer fragments [6,7]. For practicing physicians, it is of interest to study the processes of tissue reaction to the implantation of biodegradable polymers and, in general, the body’s reaction to this material [8,9]. At present, there is no single marker in medicine for assessing the body’s reaction to implantation of synthetic materials [10,11]. Attempts have been made to use C-reactive protein to assess patients’ reaction to implantation of polypropylene [12]. Currently, medical science and clinical practice rely on many inflammation markers both in tissues where inflammation occurs and in the blood. The most common markers are interferons and interleukins [7,11,13,14,15]. Different subtypes of these proteins are synthesized in the course of inflammation (bacterial, viral, etc.) by different cells and act on different target cells [14,15]. CRP is a non-specific inflammation protein which generally reflects inflammatory processes in various organs and systems [13,14]. It reflects the postoperative course during tissue reaction to surgical invasion quite well [16,17]. Its variant hs-CRP is a more sensitive inflammation marker [18,19]. Hs-CRP is widely used in clinical practice and may be suitable for assessing local non-specific intra-tissue inflammation caused by a foreign body [19].

For medical and ethical reasons, studies of tissue response to prostheses in patients are rather complicated [12]. The syndrome of autoimmune inflammation, as induced by adjuvants (ASIA), introduced into practical medicine recently, adds interest to the study of the adaptive mechanisms of the human body to surgical implants [20,21]. ASIA syndrome (Schoenefeld’s syndrome/breast implant illness) was first described in 2011 as a dysregulation of the immune system in women after surgery with silicone implants. Silicone is an adjuvant for this dysregulation. Other polymer materials in implants could also be adjuvants [20]. In this context, biodegradable polymers have an advantage over other polymers. Biopolymers “disappear” in due course and cannot be adjuvants, and therefore, they do not cause immune disorders. However, this is yet to be proven by research.

Studies of tissue reactions to implants, including biodegradable ones, are as important as the search for a marker of tissue inflammation in the blood, which was the aim of our study. The objective of this research is to study the inflammatory response by the dynamics of morphometric parameters in tissues and its effect on the level of hs-CRP in the blood of experimental animals after implantation of a biodegradable polymer.

## 2. Results

### 2.1. Tissue Response to Biopolymer (Macropreparation, Morphometry)

In animals in group I, no visible signs of pathological processes were detected after the installation of the biodegradable polymer. No animals showed suppuration or rejection of the implanted material.

When examining a macropreparation consisting of a biopolymer with adjacent tissues, a uniform white connective tissue capsule was identified along the entire perimeter of the material. No foci of infiltration or hemorrhage were observed along the perimeter or in the thickness of the matrix

**Figure 1:**
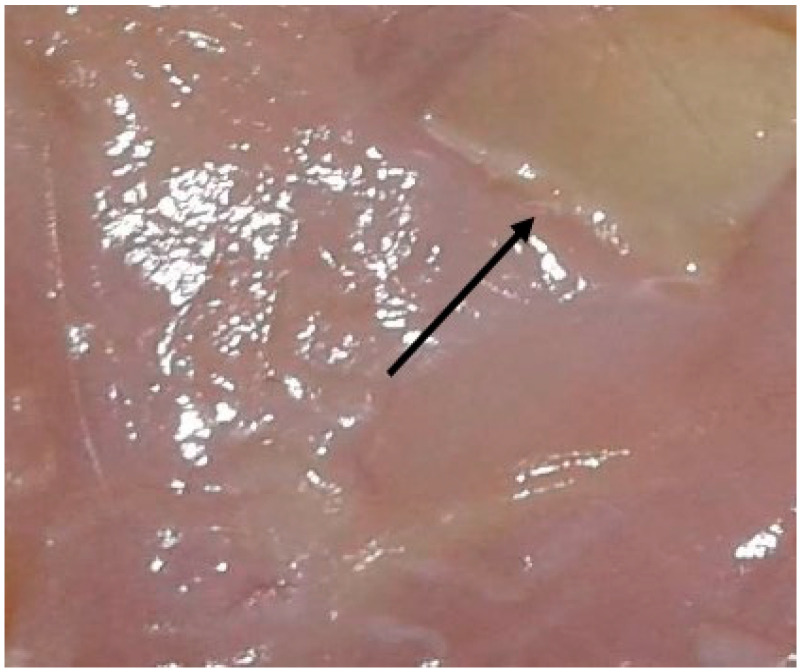
Biopolymer with clear boundaries in tissues, three months of implantation (macropreparation). Biopolymer matrix in the upper right corner, above the arrow.

With an increase in the time of withdrawal of animals from the experiment, no visible changes in the matrix size were observed; it remained the same, i.e., 1 × 1 cm. Clear boundaries of the matrix were visible for up to three months of implantation. It was impossible to determine the processes of polymer biotransformation or a decrease in its area on the macropreparation, as can be seen in Figure 1.

**Figure 2:**
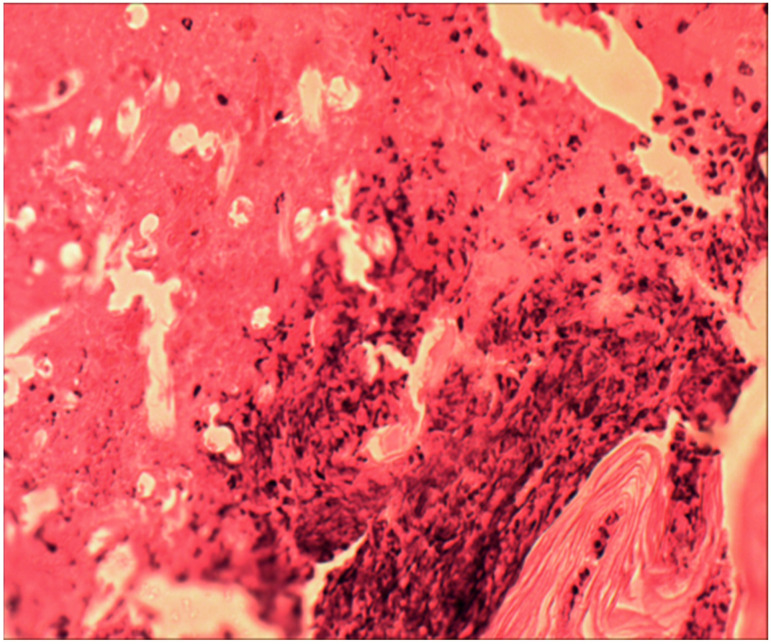
Inflammatory infiltrate around the biopolymer, second week of the experiment. The polymer border is in the lower right corner, surrounded by an infiltrate of leukocytes, macrophages, and lymphocytes. Hematoxylin and eosin staining, magnification 100×.

Under microscopy, after two weeks, taking into account the non-cellular structure of the biopolymer and its physical properties, cellular infiltration was identified only along the perimeter of the matrix; there was no migration of cells into the thickness of the material, as can be seen in Figure 2.

**Figure 3:**
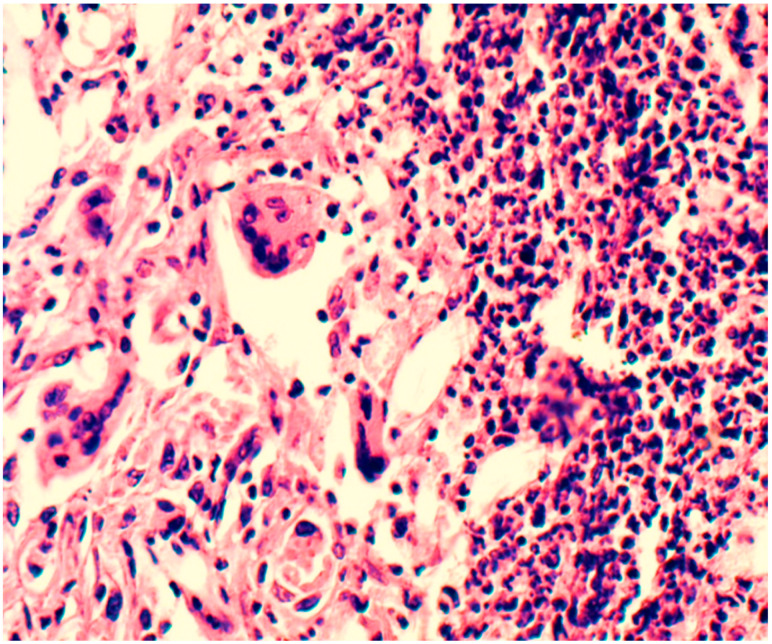
Inflammatory infiltrate around the biopolymer, second month of the experiment. The polymer border is in the upper right corner (outside the picture). In the immediate vicinity of the polymer are multiple macrophages, lymphocytes, and leukocytes. Behind them are forming vessels surrounded by fibroblasts and FBGC. Hematoxylin and eosin staining, magnification 200×.

After one month, the initial processes of cell migration into the depth of the polymer began, and collagen fibers and vessels were detected in the preparations along the periphery. Layers were distinguished in the infiltrate surrounding the material: the inner layer, closer to the synthetic material, was represented by a multitude of macrophages and leukocyte and lymphocyte cells. Further outward, single foreign-body giant cells (FBGC) were identified, behind which fibroblasts and fibrocytes were identified, as seen in Figure 3.

Lymphocytes, macrophages, and segmented leukocytes were also found in the outer layer. Collagen fibers were identified; these were evenly distributed over the entire area of the infiltrate. Elastic fibers were few and difficult to identify. In the first two weeks, on the contact line of the polymer, mainly leukocytes and lymphocytes were identified in the infiltrate; behind them were macrophages and fibroblasts. After a month of the experiment, on the border with the polymer in the infiltrate, macrophages prevailed. Leukocytes, lymphocytes, and fibrocytes were located on the second line behind the macrophages. In the following months, this picture did not change. When the biopolymer began to degrade into polymer chains (after a month), macrophages took the most active part in the absorption of these foreign chemical compounds.

After two and three months, no external signs of changes in the infiltrate surrounding the biopolymer were detected, but the area of the infiltrate increased with each month due to the advancement of granulation into the thickness of the implant.

**Figure 4:**
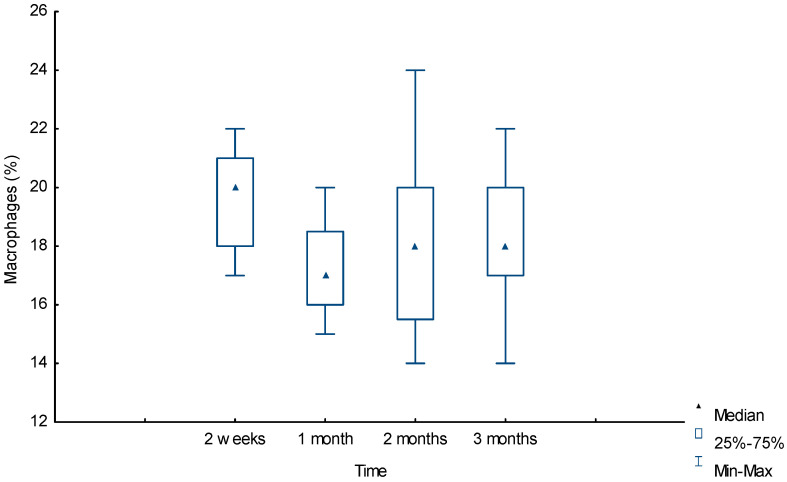
Dynamic of macrophage reaction around the biopolymer (morphometry (%) in tissues).

Figure 4 shows that when analyzing morphometric data, macrophages were one of the most numerous groups at the border with the polymer. In the first two weeks, the number of these cells was maximum at 20% [18%;21%] and significantly differed from the first-month values of 17% [16%;18.5%], U = 60.5 (*p* = 0.001) and the third-month value of 18% [17%;20%], U = 121 (*p* = 0.03), respectively. The decrease in the number of macrophages by the first month was replaced by a slight increase in the following months, without a significant difference from the first-month value of 17% [16%;18.5%] to the third-month value of 18% [17%;20%], U = 148.5 (*p* = 0.16). The number of FBGCs was insignificant; in the third month, their number was significantly greater than in the first month, 5% [4%;6%] and 4% [3%;5%], respectively, U = 115 (*p* = 0.02). The trajectory of the dynamics of the number of these cells repeated the trajectory of macrophages, which was most likely due polymer biotransformation after the first month and the absorption of polymer chains of the material by macrophages and FBGCs and, as a consequence, an increase in their number after the first month.

**Figure 5:**
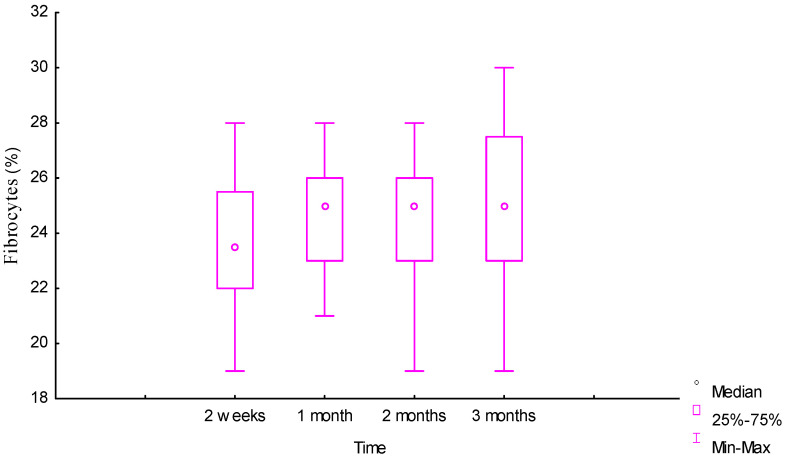
Dynamic of fibrocyte reaction around the biopolymer (morphometry (%) in tissues).

Fibrocytes and fibroblasts were massively present in the infiltrate around the biopolymer, located behind the macrophages, from as early as week 2. The trajectories of the dynamics of the number of these cells became multidirectional after two months. For fibrocytes with preservation at the same level and for fibroblasts with a slight decrease, the trajectories became multidirectional after one month 23% [22%;25%] and three months 22% [19.5%;22.5%], respectively. In general, the number of both groups of cells changed insignificantly over time, and the values for fibrocytes at two weeks and three months did not differ significantly: 23.5% [22%;25.5%] and 25% [23%;27.5%], respectively, U = 140 (*p* = 0.11), as seen in Figure 5.

**Figure 6:**
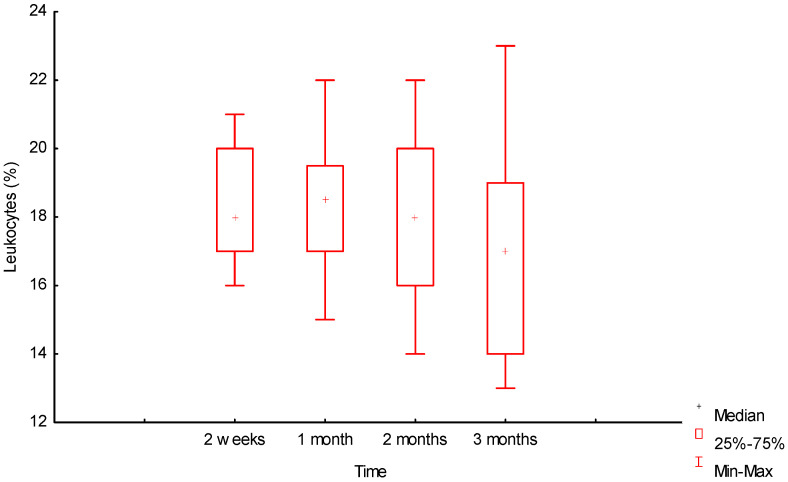
Dynamics of leukocyte reaction around the biopolymer (morphometry (%) in tissues).

**Figure 7:**
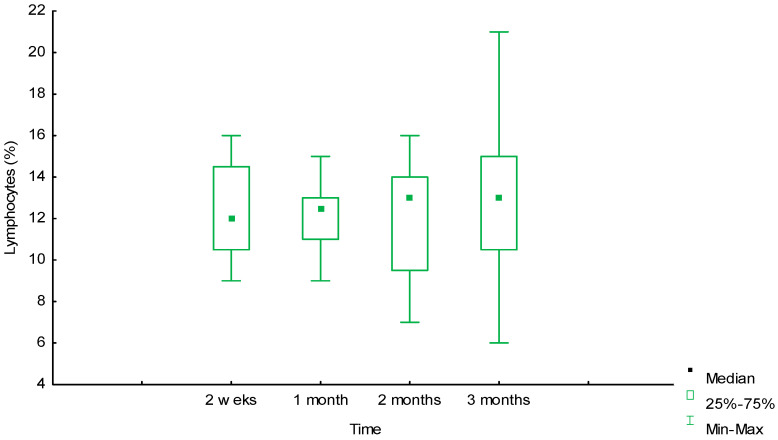
Dynamics of lymphocyte reaction around the biopolymer (morphometry (%) in tissues).

Figure 6 and Figure 7 show that the dynamics of the number of leukocytes and lymphocytes over three months was also insignificant, but in different directions, for these groups of cells.

Over the period from the second week 18% [17%;20%] to the third month 17% [14%;19%], the number of leukocytes slightly decreased (U = 128 (*p* = 0.06)), and the number of lymphocytes slightly increased 12% [10.5%;14.5%] and 13% [10.5%;15%], respectively, U = 183.5 (*p* = 0.66), but without a significant difference for both cell types. There was no significant difference in the values of the number of lymphocytes in months 1 and 3: 12.5% [11%;13%] and 13% [10.5%;15%], respectively, U = 168.5 (*p* = 0.4). No changes and a slight increase after one month in the infiltrate of this group of cells, as well as in macrophages and GCIT, was explained by prolonged inflammation in the tissues due to the advancement of the cellular infiltrate in biotransformation of the material into the depth of the biopolymer matrix.

**Figure 8:**
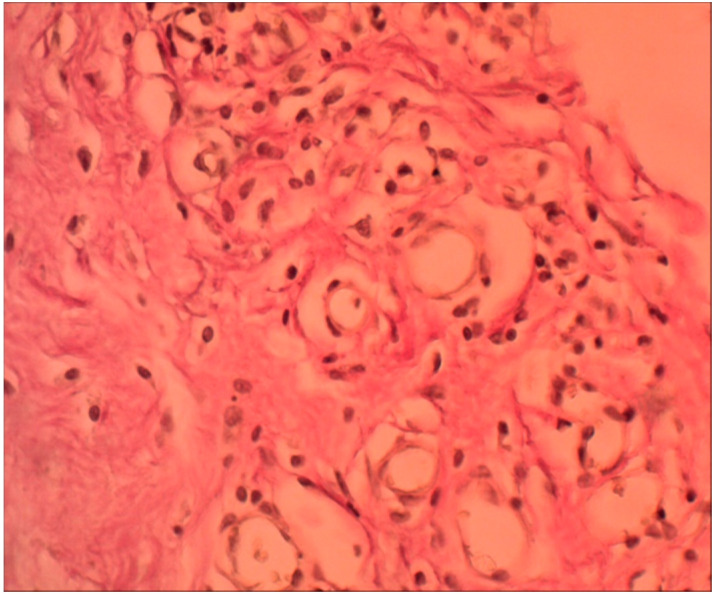
Connective tissue around degraded biopolymer, third month of the experiment. The polymer border is in the upper right corner. Closer to it is inflammatory infiltration, behind which are many newly formed vessels, fibroblasts, and fibrocytes. In the lower left corner is dense unformed connective tissue with many collagen fibers. Van Gieson staining, magnification 200×.

According to the morphometry data, after two and three months, the structure and qualitative composition of the infiltrate at the border with the biopolymer changed insignificantly, but the area of the inflammatory infiltrate increased steadily and shifted into the matrix depth. In areas distant from the biopolymer, many collagen fibers and vessels were formed, and the structure of the infiltrate acquired the form of dense unformed connective tissue, as seen in Figure 8.

### 2.2. Animal Experiment, Level of hs-CRP in Blood, ELISA

**Figure 9:**
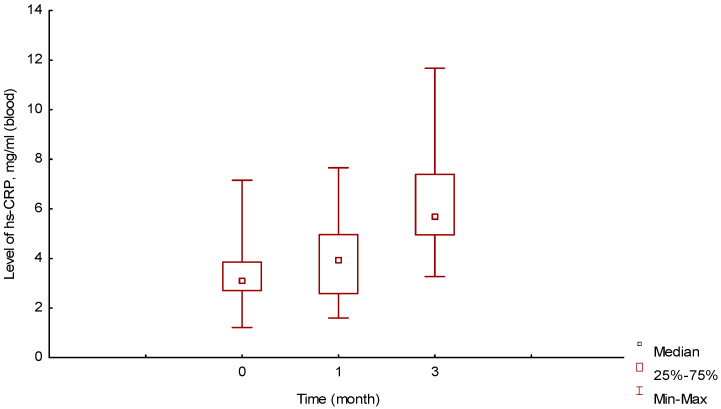
Changes in blood hs-CRP levels over time, ELISA (mg/mL).

In animals of group II, no complications were observed throughout the experiment. The median value of hs-CRP in the blood at the initial point of the experiment, before the operation, in this group of animals was 3.13 [2.7;3.85] mg/mL, as seen in Figure 9.

The average value was 3.35 ± 1.27 mg/mL, which was slightly lower than the level described in the literature for this type of intact rat [22,23].

One month of implantation, the median value of hs-CRP slightly increased to 3.92 [2.58;4.96] mg/mL and did not differ significantly from the value at the initial point (before the operation), U = 175 (*p* = 0.26).

The median value of hs-CRP in the group of animals three months after implantation increased to 5.71 [4.95;7.39] mg/mL and significantly differed from the levels at the initial point and after one month, U = 30 (*p* = 0.001) and U = 85 (*p* = 0.001), respectively.

Changes in the level of hs-CRP fully correlated with morphometric changes in the peri-implantation tissues. The intra-tissue processes influenced the increase in the level of hs-CRP at three months.

### 2.3. In Vitro/in Vivo Biopolymer Degradation, Spectrometry, and Chromatography

The results of the spectrometric analysis of the biopolymer in vitro are presented in Table 1.

According to these data, the biopolymer mostly began to degrade two months after implantation. At three months, the Mw value decreased by 14.4% compared to the initial value.

The material obtained from animal tissues after three months of implantation, according to the spectrometric data, had the following indicators: Mw = 109.5 [105;112] kDa, Mn = 66 [63;71] kDa, PDI = 1.64 [1.56;1.71].

**Figure 10:**
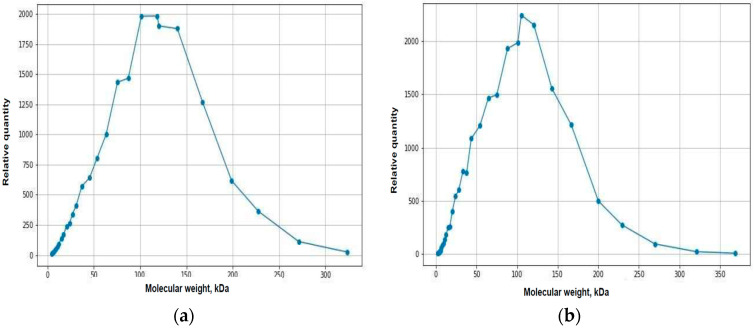

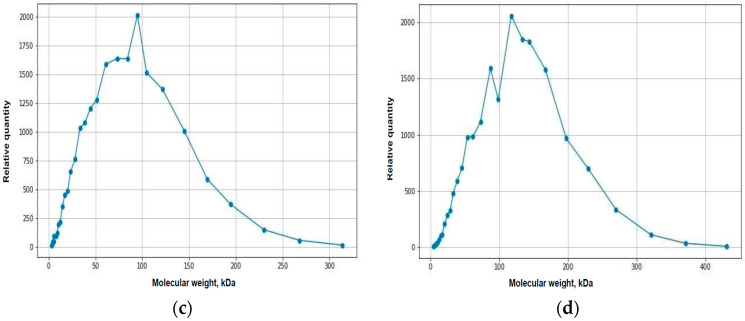
Results of biopolymer chromatography: (**a**) in vitro at 0 months; (**b**) in vitro at 2 months; (**c**) in vitro at 3 months; (**d**) in tissue, 3 months after implantation.

Figure 10 shows the graphical results of biopolymer chromatography at different times and under different conditions.

## 3. Discussion

Degradable polymers are of practical interest for surgery [3,7,24]. The study of tissue and organism response to implantation of degradable prostheses is rather complex. Physical and chemical parameters following implantation of non-degradable implants (silicone, polypropylene, metal, etc.) remain stable in the foreseeable time horizon; a limiting capsule is formed in the tissues [5,12,25,26]. When implanting a prosthesis made of a degradable material, two dynamic processes that influence each other are observed: degradation of the material, which causes physiological persistent inflammation; inflammatory response of tissues and the organism to the implant, which affects the degradation rate [2,24,26,27]. In our experiment, the interaction of the organism and the implant occurred according to this algorithm. The limiting capsule was formed only in the first month of the experiment, and then, two dynamic processes took place. Inflammation moved inside the degrading and decreasing area of the polymer matrix. Each dynamic system—organism or implant—depends on many factors.

The quantitative proportion of chemical compounds included in polymer affects the degrading ability of the material [1,6,24,27]. The initial time and rate of degradation determine the physical properties of the biopolymer, primarily its mechanical strength and, accordingly, the possibility of its application. To assess these parameters, a comparative assessment should be conducted, with degradation under standardized conditions in vitro, which can serve as the basic dynamics of polymer transformation over time [1,2,24,25,26,27]. Based on the experiment and the degradation of the biopolymer under such standardized conditions, it was found that the polymer has an average degradation rate.

There are multiple ways to alter the degradation rate, such as the application of perforations and roughness to the polymer matrix, X-ray exposure, and various methods of polymer synthesis, among others [1,4,24,25]. In the experiment, no additional methods for altering the polymer degradation rate were used. The same material was studied under different conditions in vivo and in vitro.

Implantation of a polymer material into biological tissues is also a way to affect the degradation rate of the polymer [3,5,27]. The aim of the study was not to assess the timing or the rate of degradation of a specific polymer. However, certain factors affecting the polymer degradation in tissues may be suggested as a result of the experiment. Inflammation in tissues was the main factor. In the area of inflammatory reaction, the temperature increased, the pH of the medium changed towards acidosis, the complex chemical composition of the surrounding tissue environment changed not only due to the diffusion of various free ions (Cl^−^, HCO_3_^−^, Na^+^, K^+^, Ca^2+^, and others) but also due to inflammation mediators [7,8,14,15]. Inflammatory exudate with a complex chemical composition can accelerate or slow down biodegradation, which is yet to be studied further. In the experiment, polymer degradation in vitro, according to chromatography data, began after two months, and, according to morphometry data, it began in vivo a month of implantation. Thus, degradation in tissues occurred faster than under standardized conditions.

Active phagocytosis of polymer chains in the inflammation area also clearly affects the rate of biotransformation [7,8,10]. This process can be selective with respect to specific chemical compounds: caprolactone or trimethylene carbonate. According to the results of the in vitro experiment, the proportion of these chemical compounds remained in the same proportion: 35/65%. In other words, the biopolymer degraded evenly due to both chemical compounds. The results of the polymer ratio after degradation in vivo remained beyond the scope of our experiment. This may also be the subject of future studies. Determination of the molecular weight of polymer chains during matrix degradation under standardized conditions in vitro and the correlation of these data with the ability of inflammatory cells to absorb particles of certain sizes may provide an idea of the effect of phagocytosis on the rate of biotransformation. Tissue reactivity, i.e., the expressiveness of the body’s immune response to the implantation of a foreign polymer, affects all of the above physiological factors of inflammation with a negative or positive sign. Therefore, the immune status also determines the rate of polymer biotransformation [3,7,8].

The described properties of the biopolymer and the processes occurring with it over time and under in vitro and in vivo conditions are of great scientific and practical interest. The main goal of the experiment was to study the inflammation process in peri-implantation tissues and in the blood over time and to establish potential correlation of these processes [1,2,4]. In the course of this research, we have attempted to answer the following main questions that arise when studying the use of biodegradable polymers in prostheses [24,25,26,27]:

(a) When does biodegradation begin in vivo and how does inflammation proceed in the tissues around the polymer?
(b) What is formed at the site of the degraded polymer?
(c) Is the selected biopolymer bioinert?
(d) When does biodegradation begin in vitro, and is it possible to determine the dependence on biodegradation in vivo?
(e) Can hs-CRP be used as a marker for determining the quality of inflammatory processes in peri-implant tissues?

Our research findings were as follows:

(a) Using morphometry, it was established that this polymer begins to biotransform one month after implantation, when the transformation of the synthetic matrix begins under the influence of physicochemical degradation processes and biological transformation processes, accompanied by the absorption of released biopolymer chains by macrophages. Before this period, an inflammatory infiltrate was formed along the border with the material, which after one month, due to biotransformation processes, moved into the depth of the polymer matrix, increasing the area of inflammation. In terms of qualitative composition, in the first two weeks, the inflammatory infiltrate was mainly represented by macrophages, leukocytes, and lymphocytes. After one month, the number of inflammatory cells decreased, especially macrophages—they were partially replaced by fibrocytes and fibroblasts. Subsequently, the composition of the infiltrate changed little.
(b) The result of the fibroblastic reaction was the development of dense irregular connective tissue at a distance from the border with the biopolymer after the acute phase of inflammation.
(c) The material used in the study did not cause rejection or allergic reactions in any of the cases. During the experiment, no extreme increase in the level of hs-CRP was recorded. The biopolymer consisting of PCL (65%) and PTMC (35%) can be considered bioinert.
(d) In vitro, the polymer biodegradation began from week 2. According to the spectrometry data, this process was not linear; in the first two months, the Mw and Mn values remained virtually unchanged from week 2. At three months, the Mw value was 14.4% less than the initial value. The degradation process in tissues, according to the spectrometry data during three months of implantation, remained unclear in the course of this research. The Mw and Mn values for the biopolymer from tissues after three months of implantation were greater than these values for the polymer degrading in vitro at three months and even at 0 months. Complex metabolic processes in tissues during inflammation and involving various chemical agents can explain this contradiction. The data obtained can serve as a basis for further, more detailed study of the physical parameters of polymer degradation in tissues and in vitro. However, the in vitro results confirm a delayed degradation period of at least a month, which was confirmed by the histological study of peri-implantation tissues. According to histological studies, cell migration into the biopolymer began in the first month of the experiment. This is somewhat earlier than the polymer degradation processes in vitro. As described above, during this period, macrophages were predominantly in direct contact with the biopolymer. Based on this, it can be assumed that macrophages are active participants in the biotransformation process and absorb polymer chains of the biopolymer as a result of phagocytosis. This assumption can also serve as a subject for future studies to determine the molecular weight of polymer chains formed during degradation and the ability of macrophages to absorb these chains, determining the rate of biopolymer biotransformation.
(e) Changes in the level of hs-CRP fully correlated with morphometric changes in the peri-implantation tissues (Figure 9). In the first month after implantation, the processes of adaptation of the body to the alteration due to the operation were already completed, and the process of polymer biotransformation had not yet begun; the tissue reaction was like the formation of a limiting granulation ridge with minor inflammation and a decrease in the number of macrophages, leukocytes, lymphocytes, and FBGC, which was confirmed by the absence of an increase in the level of hs-CRP in the blood of animals. After three months, the onset of the polymer biodegradation process led to an increase in the area of inflammation due to the expansion of the infiltrate into the depth of the implant with a large number of macrophages, lymphocytes, leukocytes, FBGC, and accordingly, inflammation mediators. These intra-tissue processes influenced the increase in the level of hs-CRP at three months.

Surgeons of various specialties very often use CRP as a marker of postoperative complications, most often infectious. Recently, the results of studies of the CRP level as a predictor of these complications have appeared [17,18,28]. That is, the studies record a significant increase in the CRP level on postoperative days 1, 2, and 3, when there are no clinical manifestations of infectious complications [15,29,30]. Such results confirm the high sensitivity of this inflammatory protein. Hs-CRP is also actively used as a marker of the dynamics of postoperative complications, including non-cardiological operations [19,31,32]. There is little information in the literature on the use of CRP and hs-CRP to assess adaptation of the body and tissues to the implantation of various synthetic prostheses [18,33]. Any synthetic prosthesis in biological tissues is a foreign body and causes inflammation [8,12]. The sensitivity of CRP and hs-CRP to this inflammation has been poorly studied. Even less studied is the sensitivity of CRP to the implantation of biodegradable polymers, when polymer chains separate from a foreign body over time [5,7]. The experiment confirmed the development of prolonged inflammation in tissues following implantation of the biopolymer. Based on this fact, it can be assumed that prolonged inflammation can lead to an increase in the level of CRP. Given the small size of the polymer matrix and the slow movement of inflammation into its thickness, hs-CRP was chosen in the experiment as a more sensitive inflammation protein.

The results of this experiment can be used in further studies on patients. It is highly likely that the tissue reaction in patients will be similar to that described in the study. The use of hs-CRP can also be recommended in practical medicine not only for implantable biodegradable materials but also for other prostheses made of synthetic polymers to assess the adaptation of tissues and the body to surgical intervention using prostheses. The question remains as to how much the human immune system will react to foreign synthetic polymers. To answer this question, the tissue reaction in animals and patients to widely used prostheses, for example, those made of polypropylene, may also be studied. The size of the implanted material will determine the sensitivity threshold of hs-CRP in the blood to surgical invasion. It is likely that in the case of a small implant, the area of inflammation in the tissues will also be small and will not trigger an increase in the level of inflammatory protein. The answer to this question can also be provided through animal experiments. Considering the experimental nature of the implanted material and the need for histological examination of tissues, the study was conducted on animals rather than humans. However, the results obtained may be of interest for practical medicine.

In summary, the experiment confirmed the bioinertness of the polymer from polycaprolactone and polytrimethylene carbonate (65/35%) with an average rate of biodegradation. Under in vivo conditions, degradation began after a month; under in vitro conditions, the process was slower, and degradation began after two months. Implantation of the biopolymer in tissues causes prolonged inflammation mainly due to macrophage proliferation. Over time, the inflammation increases in the area, shifting into the thickness of the polymer against the background of its decrease due to biotransformation. Unformed dense connective tissue emerges at the site of the degraded polymer after inflammation. The level of hs-CRP in the blood correlated with inflammatory activity in the tissues. This inflammatory protein can be recommended as a marker for assessing inflammation in peri-implantation tissues.

## 4. Materials and Methods

Two groups of 21 and 32 animals (laboratory white male Wistar rats aged 3 months) were implanted under the skin of the back with a biopolymer matrix (measuring 1 × 1 × 0.2 cm) consisting of 65% polycaprolactone (PCL) and 35% polytrimethylene carbonate (PTMC). The studies were carried out in accordance with the Declaration of Helsinki of the World Medical Association on the humane treatment of laboratory animals and the directive of the European Community (86/609 EU).

### 4.1. Animal Experiment, Tissue Research, and Morphometry

In group I—consisting of 32 laboratory rats—8 animals were withdrawn from the experiment after two weeks, and then, 8 more were withdrawn after 1, 2, and 3 months. Histological material was collected in the form of a biopolymer with surrounding tissues. After fixing the samples in a 10% solution of neutral formalin and standard processing on a Leica TP1020 histological complex, they were embedded in paraffin blocks. Sections 3–4 μm thick were made on a Leica RM2235 rotary microtome (Leica Biosystems, Heidelberg, Germany). Paraffin sections were stained with hematoxylin and eosin in a Leica ST5010 apparatus and according to van Gieson to identify collagen fibers (Leica Biosystems, Heidelberg, Germany). The histological preparations were examined in an Olympus CX 31 microscope (Olympus, Tokyo, Japan) with a Nikon DS-Fi1 digital video camera (Nikon, Tokyo, Japan). In the implantation zones, the formed granuloma was assessed in close proximity to the synthetic material, and the qualitative and quantitative parameters of the cellular and non-cellular components of the connective tissue forming around the implanted samples were determined. The number of neutrophilic leukocytes, fibrocytes, fibroblasts, lymphocytes, macrophages, and foreign-body giant cells (FBGCs) was counted. Cells of at least 100 were counted in the field of view at a magnification of 400×; the number of each type of cell was expressed as a percentage. The median values of the number of cells at different times were compared using the *nonparametric* Mann–Whitney *U* test. The analyzed values were measured automatically using the Bio Vision 4.0 program.

### 4.2. Animal Experiment, Study of hs-CRP, and ELISA

In group II, consisting of 21 animals, blood was collected from the animals’ tails at three timepoints: on the day of the surgery before implantation of the material; one month after implantation of the material; and three months after implantation of the material. Blood was collected in test tubes with EDTA, the tubes were centrifuged for 10 min at 400× *g*, and plasma was collected in labeled 1.5 mL tubes. Then, the samples were studied using the ELISA kit for highly sensitive C-reactive protein (hs-CRP) (Elabscience, Wuhan, China). According to the manufacturer’s instructions for the analysis, the serum was diluted 200,000 times. Spectrophotometry was performed using a Multiscan Sky device (Termo Scientific, Singapore). The hs-CRP measurements obtained at different timepoints were compared with each other by the median value to determine any significant difference using the nonparametric Mann–Whitney *U* test.

### 4.3. In Vitro Biopolymer Degradation Studies, Spectrometry, and Chromatography

To assess the time frame of the biodegradation processes in vitro, the biopolymer was studied on an Agilent 1200 ELSD chromatograph (Agilent Technologies, Santa Clara, CA, USA) to determine Mw—average molecular weight, Mn—average number molecular weight, PDI—polydispersity index (molecular weight distribution coefficient) at the following times after the manufacture: 0 days, 2 weeks, 1 month, 2 months, 3 months, 4 months, and 5 months. The actual molar ratio of PTMC and PCL units in the polymer chain (in %) was determined using a DRX-400 and AVANCE 500 nuclear magnetic resonance spectrometer, using CDCl3 as a solvent (Bruker BioSpin, Ettlingen, Germany). The polymer biodegradation outside biological tissues was carried out according to the international standard ISO 13781:2017 [34]. The biopolymer was placed in Sorensen’s buffer solution (18.2% potassium hydrogen phosphate and 81.8% sodium hydrogen phosphate), pH 7.4 ± 0.2, in a weight ratio of 1:45. The material was left in the buffer in an inert glass container at a temperature of 37.1 °C.

The tissues of six animals removed from the experiment three months after surgery were also examined using a chromatograph to determine median values of Mw, Mn, and PDI. All results were compared with the initial characteristics at 0 days.

## 5. Conclusions

The experiment confirmed the bioinertness of the complex biodegradable polymer consisting of 65% polycaprolactone and 35% polytrimethylene carbonate at the tissue and organism levels.

The level of hs-CRP repeated the dynamics of inflammation in the tissues. The absence of changes one month after the implantation against the background of a decrease in the inflammatory response in the tissues was replaced by a significant increase after three months. At this time, the area of inflammation around the implant increased, and the long-term generation of new inflammatory cells was reflected in a significant increase in the level of hs-CRP in the blood three months after implantation compared to one month after implantation and compared with the preoperative value.

Based on the results obtained, it was revealed that hs-CRP reflects tissue inflammation around the implanted material. This inflammatory protein can be recommended for assessing the adaptation processes both in tissues and in the whole organism when using synthetic biopolymers in surgical practice.

## Figures and Tables

**Table 1:** Results of biopolymer spectrometry and chromatography at different times (in vitro).

Period	The Actual Molar Ratio of Units in the Polymer Chain, %		Mw, (kDa)	Mn, (kDa)	PDI
	PTMC	PCL			
0	35	65	104.0	62.0	1.68
2 weeks	29	71	101.0	53.8	1.88
1 month	32	68	99.0	52.0	1.61
2 months	31	69	101.5	62.0	1.64
3 months	34	66	89.0	51.5	1.73
4 months	31	69	75	42.8	1.75
5 months	29	71	72.6	46.5	1.56

## Data Availability

The data that support the findings of this study are available from the corresponding author upon reasonable request.
